# Administration of zoledronic acid enhances the effects of docetaxel on growth of prostate cancer in the bone environment

**DOI:** 10.1186/1471-2407-6-15

**Published:** 2006-01-17

**Authors:** Kristen D Brubaker, Lisha G Brown, Robert L Vessella, Eva Corey

**Affiliations:** 1 Department of Biological and Allied Health Sciences, Bloomsburg University, Bloomsburg, PA, USA; 2 Department of Urology, University of Washington, Seattle, WA, USA

## Abstract

**Background:**

After development of hormone-refractory metastatic disease, prostate cancer is incurable. The recent history of chemotherapy has shown that with difficult disease targets, combinatorial therapy frequently offers the best chance of a cure. In this study we have examined the effects of a combination of zoledronic acid (ZOL), a new-generation bisphosphonate, and docetaxel on LuCaP 23.1, a prostate cancer xenograft that stimulates the osteoblastic reaction when grown in the bone environment.

**Methods:**

Intra-tibial injections of LuCaP 23.1 cells were used to generate tumors in the bone environment, and animals were treated with ZOL, docetaxel, or a combination of these. Effects on bone and tumor were evaluated by measurements of bone mineral density and histomorphometrical analysis.

**Results:**

ZOL decreased proliferation of LuCaP 23.1 in the bone environment, while docetaxel at a dose that effectively inhibited growth of subcutaneous tumors did not show any effects in the bone environment. The combination of the drugs significantly inhibited the growth of LuCaP 23.1 tumors in the bone.

**Conclusion:**

In conclusion, the use of the osteolysis-inhibitory agent ZOL in combination with docetaxel inhibits growth of prostate tumors in bone and represents a potential treatment option.

## Background

Patients with advanced prostate cancer (CaP) suffer from the severe consequences of hormone-refractory disease and bone metastases. After development of metastatic hormone-refractory disease, CaP is incurable, with a median survival of 9 to 12 months. Given the near-certainty that processes associated with hormone-refractory CaP and bone metastasis contribute directly to morbidity (pain, bone fractures, bone marrow failure) of patients, treatment modalities that would eliminate the advanced disease, slow down progression, or improve the quality of life of patients with advanced CaP are of great interest. Hormonal therapy, radiotherapy, and chemotherapy do not cure hormone-refractory disease [1]. Attacking the tumor cells by multiple mechanisms may prove to be much more effective in killing tumor cells; therefore combined chemotherapies are one of the most promising themes of today's clinical picture.

Zoledronic acid (ZOL) is one of the most potent of the new-generation bisphosphonates (BPs), compounds that inhibit bone lysis and have been used in the treatment of bone diseases and bone metastastatic disease [2-7], and stimulate apoptosis in cancer cells [8-10]. Effects of ZOL on tumor cells [11,12], including prostate tumors [13-19], have been reported in the literature. In our previous *in vivo *studies we have shown that growth of subcutaneous CaP tumors was not inhibited by ZOL; however, growth of prostate cancer cells in the bone environment was significantly inhibited, but the tumors were not eradicated [15]. We have also shown that the probable mechanisms of the effects of ZOL on tumor in bone are indirect, *via *its effects on bone cells [19]. The value and potential of ZOL in treatment of patients with cancer-related bone disease were reviewed [5], and a large number of reports have recently documented the benefits of ZOL treatment in patients with advanced prostate cancer [20-31]. However, in parallel with our pre-clinical study, these published results suggest that BPs can slow progression of the disease, but a cure is not achieved. Therefore, combinations of BPs with other agents such as chemotherapeutic drugs to control tumor growth while regulating tumor-induced bone remodeling have appeared as a promising new treatment strategy.

Docetaxel, an inhibitor of tubulin depolymerization, has emerged as the most valuable chemotherapeutic treatment for advanced CaP. Phase II studies have shown decreased prostate specific antigen (PSA) levels and increased survival, with dosage amounts and regimens varying from 25–75 mg/m^2 ^every 1 or 3 weeks [32-37]. However, the toxicity of this treatment is significant and complete cures are still not achieved [34].

The recent history of chemotherapy has shown that with difficult disease targets, combinatorial therapy frequently offers the best chance of a cure. Therefore testing of combinations of BPs with other agents is of significant interest in the treatment of various cancers. Many of the initial studies of BPs in combination with chemotherapy were conducted on breast cancer or myeloma, where the bone lesions are highly osteolytic. Combinations of taxoids with BPs *in vitro *exhibited additive anti-tumor effects against invasion and adhesion [38] and induction of apoptosis in breast-cancer cells *in vitro *[39] and *in vivo *[40]. Moreover, inclusion of BPs in standard chemotherapy has led to a sustained reduction in skeletal complications in breast-cancer patients [41-43]. In advanced CaP patients, ZOL in combination with docetaxel and low-dose estramustine phosphate was recently shown to decrease serum PSA and pain levels [34]. Additionally, Vordos *et al*. reported that ZOL in combination with docetaxel decreased serum PSA by over 50% at 2 months in more than half of the patients [25]. It has also been reported that the alendronate in combination with taxol is more effective in preventing development of bone metastases in a CaP animal model than either therapy independently [44].

In this study we used a combination of ZOL and docetaxel to evaluate changes in bone remodeling and tumor growth of an osteoblastic CaP xenograft, LuCaP 23.1.

## Methods

### Xenograft

All procedures were performed in compliance with the University of Washington Institutional Animal Care and Use Committee and NIH guidelines. LuCaP 23.1 is a PSA-producing human CaP xenograft [45], which is maintained subcutaneously (SC) in male athymic mice.

### Intra-tibial tumors and docetaxel and zoledronic acid treatment

The right tibiae of forty male SCID mice (four-to-six-week-old, Fox Chase SCID mice, Charles River, Wilmington, MA) were injected with 2 × 10^5 ^LuCaP 23.1 cells as we have described previously [15]. Briefly, 1–2 × 10^5 ^separated single LuCaP 23.1 cells in ~10–20 μl were injected into the proximal end of the tibia. After the procedures, animals received Buprenorphine as an analgesic (2 mg/kg body weight subcutaneously) until fully recovered (usually <24 hours). The tumor growth was monitored by measurements of serum prostate specific antigen (PSA) levels. The animals were randomized into 4 groups when tumors in bone were established (mean serum levels of PSA: 5–10 ng/ml). Groups were as follows: 1) a control group which received subcutaneous PBS injections; 2) a ZOL treatment group which received subcutaneous injections of ZOL twice a week (2.5 μg per injection, ~0.1 mg/kg, Novartis Pharma AG, Basel, Switzerland.); 3) a docetaxel treatment group which received intra-peritoneal injections of docetaxel every two weeks (400 μg per injection, 20 mg/kg, Aventis Pharmaceuticals Inc. Bridgewater, NJ); and 4) a group treated with a combination of ZOL + docetaxel which received subcutaneous injections of ZOL twice a week (2.5 μg per injection, ~0.1 mg/kg) and intra-peritoneal injections of docetaxel every two weeks (400 μg per injection, 20 mg/kg). Docetaxel was dissolved in ethanol and polysorbate 80. The final dilutions were prepared just before injection with 5% dextrose. Blood samples were collected every week for determination of serum PSA levels (IMx Total PSA assay, Abbott Laboratories, Abbott Park, IL). The mice were sacrificed 7 weeks after enrolment. Bone mineral density (BMD) was measured prior to sacrifice in the area adjacent to the growth plate (PIXImus Lunar densitometer, GE Healthcare, Waukesha, WI). Radiographs of the tibiae were taken prior to sacrifice. At sacrifice, five tumored tibiae from each group were embedded in methacrylate for bone histomorphometrical (BHM) analysis.

### Bone histomorphometry

BHM analysis was performed on 6-μm longitudinal sections of calcified tibiae stained with Goldner stain in the middle of the tibia from five animals per group (Skeletech, Inc., Bothell, WA). The percentages of bone volume and tumor volume in the whole longitudinal section of tibiae (% BV/TV and % TuV/TV, respectively) were calculated. Detailed analysis of the trabecular bone, including determination of trabecular thickness in μm (Tb.Th.), trabecular number per mm (Tb.N.), trabecular separation in μm (Tb.Sp.), the ratio of osteoblast surface to bone surface as a percentage, the ratio of osteoclast number to bone surface as a percentage, osteoid surface to bone surface as a percentage (Os.S/BS) and osteoid thickness (Os.Th.), was performed in the area adjacent to the growth plate (0.525–1.225 mm below the growth plate, site of injection). Statistical analysis of the results was done using paired and unpaired Student's t tests as appropriate.

### Effects of docetaxel on subcutaneous tumors

Six-to-eight-week-old male SCID mice were implanted with LuCaP 23.1 tumor fragments over the right scapular region. Tumor volume was measured twice weekly and calculated as L × H × W × 0.5236. After tumors reached 200 mm^3 ^in size, animals were randomized into two groups. Group 1: animals received intra-peritoneal injections of PBS every two weeks; Group 2: animals received intra-peritoneal injections of 20 mg/kg docetaxel (Bristol-Myers Squibb, Princeton, NJ) every two weeks. Tumor growth was monitored twice weekly. Animals were sacrificed when the tumors reached ~1000 mm^3 ^or when animals were becoming compromised.

## Results

**Figure 1:**
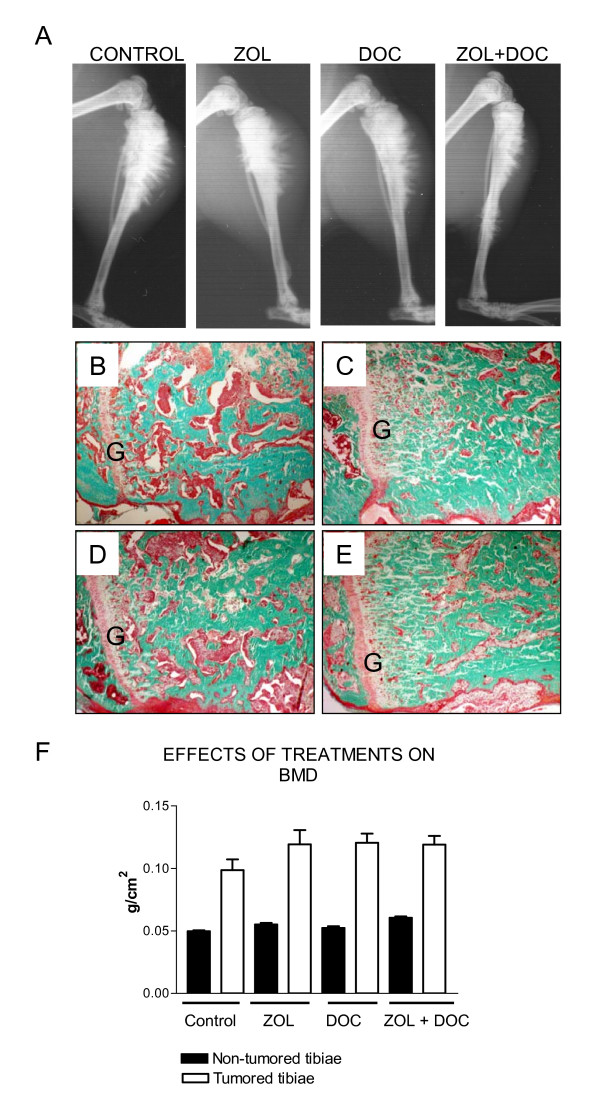
**A. Radiographs of treated and untreated LuCaP 23.1 bone tumors**. Extensive new bone formation stimulated by LuCaP 23.1 is visible in the control tibiae. Although substantial bone formation is also visible with ZOL and ZOL + docetaxel, these tibiae exhibit decreased periosteal activity compared to the control and docetaxel-treated tibiae. **B.-E. Histological appearance of treated and untreated LuCaP 23.1 bone tumors**. Sections stained with Goldner reagent show marrow or tumored areas in pink and bone in green. The growth plate is marked with the letter G. The ZOL- (C) and ZOL + docetaxel-treated groups (E) exhibit substantial increases in bone volume and decreases in tumor volume compared to the control (B) and docetaxel-treated (D) groups. **F. Bone mineral density (BMD) measurements at sacrifice**. BMD was measured for the non-tumored and tumored legs of all four treatment groups. BMD was higher in tumored tibiae than in non-tumored tibiae, but there were no significant differences among the four treatment groups.

LuCaP 23.1 causes significant new bone formation with a pronounced periosteal reaction. Radiographs of ZOL-treated tibiae show denser bone with a smaller perimeter. Importantly the tibiae treated with the combination of ZOL and docetaxel show dense bone and decreases in the periosteal reaction. Therefore the radiographs demonstrate a decrease in the overall extensive periosteal bone formation observed with the LuCaP 23.1 intra-tibial tumors by both ZOL alone and the combination of ZOL + docetaxel. Representative examples of the radiographic appearance of tibiae from control and treated animals are presented in Figure 1A. Representative examples of the histological appearance of control and treated tibiae are shown in Figures 1B–E. The decrease in tumor volume (red/pink stain) and increase in bone volume (green stain) upon treatment with ZOL alone (Figure 1C) or the ZOL + docetaxel combination (Figure 1E) can be clearly seen in the comparison with the control animal (Figure 1B). The bone in the animals treated with ZOL appears somewhat more "organized" in the areas adjacent to the growth plate. This effect may be due to the larger volume of bone in this area (data not shown).

Effects of osteoblastic CaP xenograft LuCaP 23.1 and ZOL and docetaxel on bone were further evaluated by measuring BMD. BMD of all tumored tibiae exhibited increased BMD in comparison to the non-tumored tibia (196–240% increases, p < 0.0001 for all, Figure 1F). These increases are due to the stimulation of osteoblastic activity by LuCaP 23.1 and, in groups 2 and 4, by administration of ZOL. However, no significant differences in BMD of tumored tibiae were observed among the four groups. The systemic effect of ZOL was demonstrated by low but statistically significant increases in the BMD of non-tumored tibiae in groups 2 and 4 compared to the normal tibiae of group 1 (group 1: 0.0496 ± 0.008 g/cm^2^, group 2: 0.0551 ± 0.001 g/cm^2 ^(111%), and group 4: 0.0605 ± 0.001 g/cm^2 ^(122%), p < 0.002 for both).

We next performed a more detailed analysis of the effects of the treatments on bone, using bone histomorphometry. Groups receiving ZOL (2 and 4) exhibited 58% and 75% increases in bone %BV/TV over the control group (p = 0.0836 and p = 0.0164). These results are consistent with the histological appearance of the tibiae (Figures 1B–E). LuCaP 23.1 and ZOL both caused increases in bone volume; therefore we cannot determine with certainty the source of the increases in bone volume – LuCaP 23.1., ZOL alone, or the reduction in volume of the tumors combined with the anti-resorptive properties of ZOL. In our study the %BV/TV did not correlate with BMD values. This discordance may be due to potential effects of the treatments on mineral deposition or the methodology used. Trabecular numbers and Tb.Sp. were similar in all 4 groups, with a trend to increased Tb.Th in group 2, which received ZOL alone (group 2: p = 0.0705). Os.S/BS was decreased in ZOL-treated animals by 35–45%, but these differences were not statistically significant, and no differences were detected in Os.Th. between the groups. ZOL administration resulted in decreased numbers of osteoclasts (Table 1, group 1 *vs*. group 2: p = 0.0092; group 1 *vs*. group 4: p = 0.0030). Osteoblast perimeter was not significantly altered by any of the treatments.

**Figure 2:**
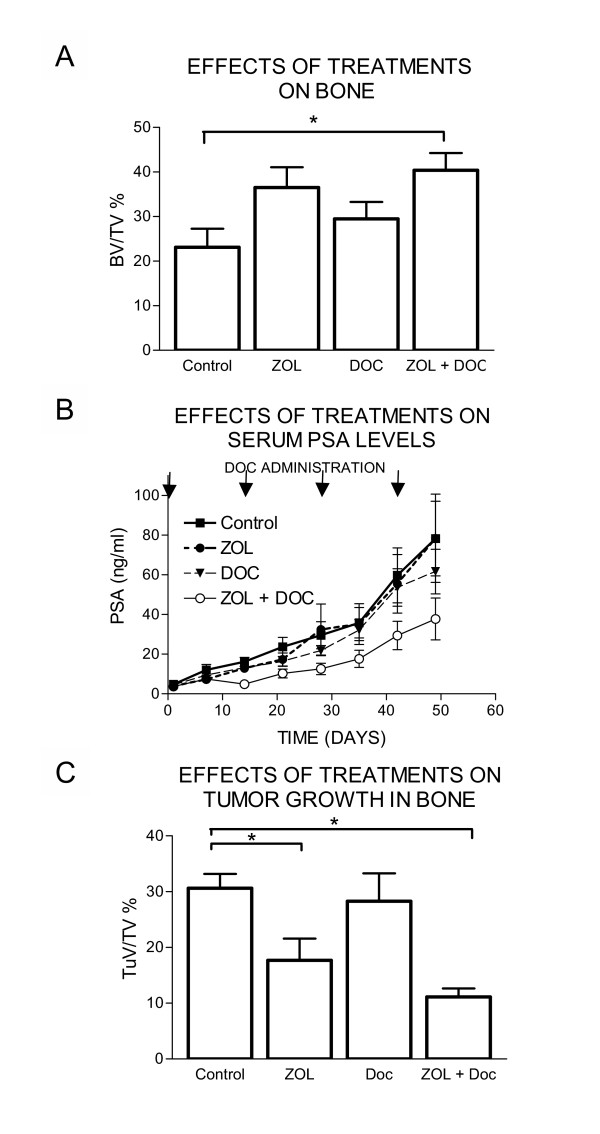
**A. Effects of ZOL and docetaxel treatments on bone volume**. Bone volume as a percentage of the total volume was determined by bone histomorphometrical analysis. The ZOL + docetaxel treatment significantly increased bone volume compared to that of the control group (p = 0.0164, *). %BV/TV of the ZOL group was increased in comparison to the control group but the changes did not reach significance (p = 0.0839). **B. Effects of ZOL and docetaxel treatments on serum PSA levels**. Blood was drawn weekly from each treatment group, control (■), ZOL (●), Docetaxel (▼), or ZOL + docetaxel (○), and analyzed for PSA levels (ng/ml). The combination of ZOL + docetaxel decreased PSA levels significantly compared to those in the control animals. **C. Effects of ZOL and docetaxel treatments on tumor volume**. Tumor volume as a percentage of the total volume was determined by bone histomorphometrical analysis for the four treatment groups. The ZOL and ZOL + docetaxel treatments decreased tumor volume significantly (p = 0.027, 0.0002, respectively *) compared to that of the control group. The combination of ZOL and docetaxel resulted in decreased tumor volume in comparison to animals receiving ZOL alone; however, these results did not reach significance (p = 0.111).

An important goal of our study was to determine the effects of ZOL and docetaxel and their combination on tumor growth. Tumor growth during the course of the treatments was monitored by PSA serum levels. Administration of ZOL and docetaxel alone did not decrease serum PSA levels in comparison to the control group (Figure 2B). However, the combination of these two agents decreased serum PSA levels to approximately 44% of that of the control animals (p < 0.05). We measured tumor volume at the time of sacrifice using BHM analysis. Administration of ZOL alone significantly decreased tumor volume in comparison to the control group (100.0 ± 8.4% *vs*. 57.7 ± 12.8%, p = 0.027). Administration of docetaxel alone at the dose used was not effective in tumor inhibition (92.4 ± 16.2%, p = 0.689). Importantly the combination of ZOL and docetaxel decreased tumor growth in comparison to control animals to 36.4 ± 5.00%, p = 0.0002. Administration of docetaxel in combination with ZOL resulted in decreased tumor volume in comparison to animals receiving ZOL alone; however, these results did not reach significance (63.0 ± 8.6%, p = 0.111).

**Figure 3:**
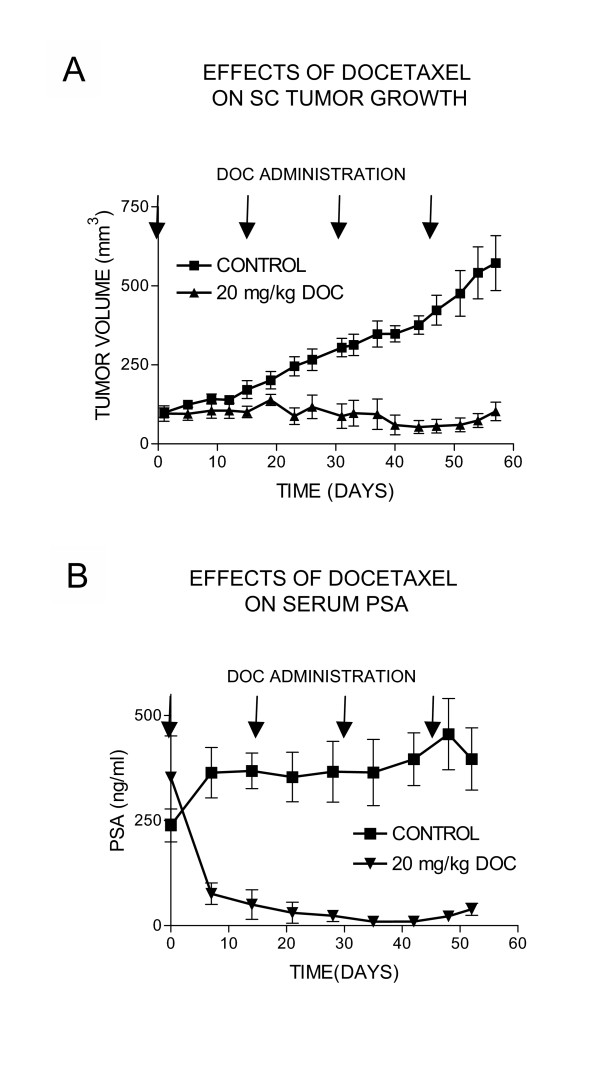
**Effects of docetaxel on growth of subcutaneous LuCaP 23.1**. LuCaP 23.1 fragments were implanted SC and animals were treated with 20 mg/kg docetaxel or placebo. Tumor volume was measured twice a week and blood was drawn weekly for determination of serum PSA levels. Mean ± SEM is plotted.**A. Tumor volume**: Docetaxel administration significantly decreased tumor volume compared to that of the control group. **B. Serum PSA levels**: Docetaxel decreased PSA levels significantly compared to those in the control animals.

We have also evaluated the effects of 20 mg/kg docetaxel on growth of subcutaneous LuCaP 23.1 tumors. This dose was effective in inhibiting growth of subcutaneous LuCaP 23.1 (Figure 3A). Serum PSA levels were also significantly decreased in animals who received docetaxel treatment (Figure 3B). Decreases in serum PSA levels were in concordance with tumor volume.

## Discussion

CaP is an extremely heterogeneous disease, and to date treatment strategies with single agents have failed to cure advanced prostate cancer. New multimodality therapeutic combinations that target different pathways and produce pronounced and sustained clinical activity without severe toxicity are now considered the next frontier in treating advanced CaP. However, as expected, not all combinations will be superior to monotherapies; *e.g*., combinations of ZOL with Imatib mesylate (Gleevec) [46] or high doses of calcitriol and dexamethasone [27] are reported to have no beneficial effects beyond those of monotherapy.

ZOL has recently emerged as an effective treatment for inhibition of both normal and pathologic bone resorption, and this has had a major impact on the treatment of metastases to the bone. In advanced CaP, ZOL has been shown to significantly reduce bone pain and the incidence of skeletal complications [47]. We have shown previously that ZOL inhibits growth of osteoblastic LuCaP 23.1 in the bone environment [15] and our new data show that lower amounts of ZOL (0.1 mg/kg) are also effective in suppression of LuCaP 23.1 growth in bone. In an earlier article we reported lowering of PSA levels as well as tumor volume by ZOL [15]. The difference may be due to the lower dosage of ZOL used in the current study. Docetaxel-induced alterations in serum PSA levels followed tumor volume in animals harboring subcutaneous LuCaP 23.1, whereas ZOL treatment of LuCaP 23.1 in bone decreased tumor volume, but changes in serum PSA were not significant. We hypothesize that PSA may not reflect changes in the response of CaP bone metastases to ZOL treatment, and that regulation of tumor growth and of PSA expression are affected by different signaling pathways and to different extents in the bone environment.

Although the main action of ZOL is inhibition of osteoclastic bone resorption [19], there is some evidence suggesting direct anti-tumor effects of ZOL as well as a possible synergism with taxanes [9, 17, 48]. Taxanes have been shown to be effective against osteolytic experimental CaP metastases [49, 50] and docetaxel has recently been approved as a standard chemotherapy for advanced prostate cancer. In the present study, 20 mg/kg docetaxel administered every two weeks significantly inhibited the growth of subcutaneous LuCaP 23.1. However, the effectiveness of docetaxel as a single drug against growth of LuCaP 23.1 in the bone was insignificant. We speculate that the bioavailability of docetaxel may be reduced in the osteoblastic environment.

In the present study, administration of ZOL apparently had the effect of sensitizing the target cells to the effects of docetaxel. In fact in the presence of ZOL, the additional effect of docetaxel (~37% reduction in growth) was almost as great as that seen with ZOL compared with the control group (~42% reduction), although it should be noted that the comparison between ZOL and the ZOL/docetaxel combination failed to reach statistical significance because of modestly higher scatter within the ZOL-only group. Similar results were recently published by Kim *et al*. [50], who reported that ZOL and paclitaxel each inhibited tumor growth in models of experimental osteolytic bone metastases of CaP, and the inhibition was even more pronounced with the combination. Studies employing combinations of agents in androgen-independent preclinical models are also needed to extend these observations to a setting more similar to advanced CaP patients.

## Conclusion

Our data show that the combination of ZOL and docetaxel is effective in inhibiting growth of osteoblastic metastases of prostate cancer. Because of the toxicity of chemotherapeutic agents such as docetaxel, it is important to insure that they are effective against disease as administered. Our data indicate that the single-agent effectiveness of docetaxel in the osteoblastic CaP environment may be limited, but ZOL may sensitize the target cells to the effects of docetaxel. These data support clinical evaluation of this combination in CaP patients.

## Abbreviations

CaP: prostate cancer, ZOL: zoledronic acid, DOC: docetaxel, BPs: bisphosphonates, SC: subcutaneously, BMD: bone mineral density, BHM: bone histomorphometry, BV/TV: bone volume in tissue volume, TuV/TV: tumor volume in tissue volume, Tb.Th.: trabecular thickness, Tb.N.: trabecular number, Tb.Sp.: trabecular separation, Ob.Pm./BPm.: osteoblast perimeter per bone perimeter, N.Oc./BS: osteoclast number per bone surface, Os.S/BS: osteoid surface per bone surface, Os.Th.: osteoid thickness

## Competing interests

The author(s) declare that they have no competing interests. Studies were supported by a grant from Novartis Pharma AG, Basel, Switzerland.

## Authors' contributions

KDB analyzed the results, and prepared the manuscript for submission;

LGB was involved in design of the studies and perform tissue culture;

RLV was involved in design of animal studies and discussion of the results;

EC was involved in design of animal studies, analyses of the data and their interpretation as well as writing the manuscript.

## Pre-publication history

The pre-publication history for this paper can be accessed here:



## Figures and Tables

**Table 1:** Results of histomorphometrical analysis of effects of the treatments on LuCaP 23.1 and bone

	**BV/TV (%)**	**TUV/TV (%)**	**TB.TH. (μM)**	**TB. N. (MM^-1^)**	**TB.SP. (μM)**	**OS.S./BS (%)**	**OS.TH. (μM)**	**OB.PM./BPM. (%)**	**N.OC./BS (%)**
**Control**	23.09 ± 9.34	30.62 ± 5.75	94.89 ± 31.75	6.06 ± 1.41	76.87 ± 20.90	7.24 ± 1.82	4.57 ± 0.29	15.97 ± 5.08	1.91 ± 0.35
**ZOL**	36.51 ± 7.86	17.68 ± 6.79^a^	145.7 ± 31.64	5.08 ± 0.53	52.29 ± 13.51	3.96 ± 1.87	5.27 ± 0.45	8.46 ± 1.42^a^	0.83 ± 0.46^a^
**DOC**	29.46 ± 8.56	28.29 ± 11.18	81.53 ± 20.45	5.61 ± 0.90	100.6 ± 24.88	6.73 ± 1.84	5.21 ± 0.36	14.17 ± 4.36	1.19 ± 0.61
**ZOL + DOC**	40.38 ± 8.73^a^	11.13 ± 3.41^a,b^	118.5 ± 25.56	5.59 ± 0.51	61.48 ± 21.83	4.77 ± 1.01	4.98 ± 0.40	11.92 ± 5.90	0.77 ± 0.42^a^

Tumored tibiae were harvested after seven weeks of treatment and embedded in methacrylate. Analysis was performed on Goldner-stained longitudinal sections of tibiae (n = 5 per group).

Results are presented as a mean ± SD.

^a ^p < 0.5 *vs*. control group

^b ^p = 0.11 group 2 *vs*. group 4

## References

[B1] Hellerstedt BA, Pienta KJ (2002). The current state of hormonal therapy for prostate cancer. CA Cancer J Clin.

[B2] Green JR (2001). Chemical and biological prerequisites for novel bisphosphonate molecules: results of comparative preclinical studies. Semin Oncol.

[B3] Major P, Lortholary A, Hon J, Abdi E, Mills G, Menssen HD, Yunus F, Bell R, Body J, Quebe-Fehling E, Seaman J (2001). Zoledronic acid is superior to pamidronate in the treatment of hypercalcemia of malignancy: a pooled analysis of two randomized, controlled clinical trials. J Clin Oncol.

[B4] Flanagan AM, Chambers TJ (1991). Inhibition of bone resorption by bisphosphonates: interactions between bisphosphonates, osteoclasts, and bone. Calcif Tissue Int.

[B5] Fleisch H (2001). Zoledronic acid: an evolving role in the treatment of cancer patients with bone disease. Semin Oncol.

[B6] Morton AR, Cantrill JA, Pillai GV, McMahon A, Anderson DC, Howell A (1988). Sclerosis of lytic bone metastases after disodium aminohydroxypropylidene bisphosphonate (APD) in patients with breast carcinoma. BMJ.

[B7] Hughes DE, Wright KR, Uy HL, Sasaki A, Yoneda T, Roodman GD, Mundy GR, Boyce BF (1995). Bisphosphonates promote apoptosis in murine osteoclasts in vitro and in vivo. J Bone Miner Res.

[B8] Shipman CM, Rogers MJ, Apperley JF, Russell RG, Croucher PI (1997). Bisphosphonates induce apoptosis in human myeloma cell lines: a novel anti-tumour activity. Br J Haematol.

[B9] Senaratne SG, Pirianov G, Mansi JL, Arnett TR, Colston KW (2000). Bisphosphonates induce apoptosis in human breast cancer cell lines. Br J Cancer.

[B10] Mackie PS, Fisher JL, Zhou H, Choong PF (2001). Bisphosphonates regulate cell growth and gene expression in the UMR 106-01 clonal rat osteosarcoma cell line. Br J Cancer.

[B11] Peyruchaud O, Winding B, Pecheur I, Serre CM, Delmas P, Clezardin P (2001). Early detection of bone metastases in a murine model using fluorescent human breast cancer cells: application to the use of the bisphosphonate zoledronic acid in the treatment of osteolytic lesions. J Bone Miner Res.

[B12] Mundy GR, Yoneda T, Hiraga T (2001). Preclinical studies with zoledronic acid and other bisphosphonates: impact on the bone microenvironment. Semin Oncol.

[B13] Lee MV, Fong EM, Singer FR, Guenette RS (2001). Bisphosphonate treatment inhibits the growth of prostate cancer cells.. Cancer Res.

[B14] Lee YP, Schwarz EM, Davies M, Jo M, Gates J, Zhang X, Wu J, Lieberman JR (2002). Use of zoledronate to treat osteoblastic versus osteolytic lesions in a severe-combined-immunodeficient mouse model. Cancer Res.

[B15] Corey E, Brown LG, Quinn JE, Poot M, M.P R, Higano CS, R.L. V (2003). Zoledronic acid exhibits inhibitory effects on osteoblastic and osteolytic metastases of prostate cancer. Clin Cancer Res.

[B16] Tenta R, Tiblalexi D, Sotiriou E, Lembessis P, Manoussakis M, Koutsilieris M (2004). Bone microenvironment-related growth factors modulate differentially the anticancer actions of zoledronic acid and doxorubicin on PC-3 prostate cancer cells. Prostate.

[B17] Coxon JP, Oades GM, Kirby RS, Colston KW (2004). Zoledronic acid induces apoptosis and inhibits adhesion to mineralized matrix in prostate cancer cells via inhibition of protein prenylation. BJU Int.

[B18] Montague R, Hart CA, George NJ, Ramani VA, Brown MD, Clarke NW (2004). Differential inhibition of invasion and proliferation by bisphosphonates: anti-metastatic potential of Zoledronic acid in prostate cancer. Eur Urol.

[B19] Quinn JE, Brown LG, Zhang J, Keller ET, Vessella RL, Corey E (2005). Comparison of Fc-osteoprotegerin and zoledronic acid activities suggests that zoledronic acid inhibits prostate cancer in bone by indirect mechanisms. Prostate Cancer Prostatic Dis.

[B20] Berenson JR (2005). Recommendations for zoledronic Acid treatment of patients with bone metastases. Oncologist.

[B21] Vogel CL, Yanagihara RH, Wood AJ, Schnell FM, Henderson C, Kaplan BH, Purdy MH, Orlowski R, Decker JL, Lacerna L, Hohneker JA (2004). Safety and pain palliation of zoledronic acid in patients with breast cancer, prostate cancer, or multiple myeloma who previously received bisphosphonate therapy. Oncologist.

[B22] Price N (2004). Benefit of extended zoledronate therapy for patients with bone metastases from hormone-refractory prostate cancer. Clin Prostate Cancer.

[B23] Parker CC (2004). Re: Long-term efficacy of zoledronic acid for the prevention of skeletal complications in patients with metastatic hormone-refractory prostate cancer. J Natl Cancer Inst.

[B24] Altundag K, Altundag O, Morandi P, Gunduz M (2004). High-dose calcitriol, zoledronate, and dexamethasone for the treatment of progressive prostate carcinoma. Cancer.

[B25] Vordos D, Paule B, Vacherot F, Allory Y, Salomon L, Hoznek A, Yiou R, Chopin D, Abbou CC, de la TA (2004). Docetaxel and zoledronic acid in patients with metastatic hormone-refractory prostate cancer. BJU Int.

[B26] Higano CS (2004). Understanding treatments for bone loss and bone metastases in patients with prostate cancer: a practical review and guide for the clinician. Urol Clin North Am.

[B27] Winquist E, Berry S (2004). Re: A Randomized, placebo-controlled trial of zoledronic acid in patients with hormone-refractory metastatic prostate carcinoma. J Natl Cancer Inst.

[B28] Saad F (2005). Clinical benefit of zoledronic acid for the prevention of skeletal complications in advanced prostate cancer. Clin Prostate Cancer.

[B29] Parker CC (2005). The role of bisphosphonates in the treatment of prostate cancer. BJU Int.

[B30] Green JR (2005). Skeletal complications of prostate cancer: pathophysiology and therapeutic potential of bisphosphonates. Acta Oncol.

[B31] Bertelli G, Heouaine A, Arena G, Botto A, Garrone O, Colantonio I, Occelli M, Fea E, Giubergia S, Merlano M (2005). Weekly docetaxel and zoledronic acid every 4 weeks in hormone-refractory prostate cancer patients. Cancer Chemother Pharmacol.

[B32] Ferrero JM, Foa C, Thezenas S, Ronchin P, Peyrade F, Valenza B, Lesbats G, Garnier G, Boublil JL, Tchiknavorian X, Chevallier D, Amiel J (2004). A weekly schedule of docetaxel for metastatic hormone-refractory prostate cancer. Oncology.

[B33] Efstathiou E, Bozas G, Kostakopoulos A, Kastritis E, Deliveliotis C, Antoniou N, Skarlos D, Papadimitriou C, Dimopoulos MA, Bamias A (2005). Combination of docetaxel, estramustine phosphate, and zoledronic acid in androgen-independent metastatic prostate cancer: efficacy, safety, and clinical benefit assessment. Urology.

[B34] Tiersten AD, Nelsen C, Talbot S, Vahdat L, Fine R, Troxel A, Brafman L, Shriberg L, Antman K, Petrylak DP (2003). A phase II trial of docetaxel and estramustine in patients with refractory metastatic breast carcinoma. Cancer.

[B35] Oudard S, Carpentier A, Banu E, Fauchon F, Celerier D, Poupon MF, Dutrillaux B, Andrieu JM, Delattre JY (2003). Phase II study of lonidamine and diazepam in the treatment of recurrent glioblastoma multiforme. J Neurooncol.

[B36] Higano CS (2005). Current status of treatment for patients with metastatic prostate cancer. Can J Urol.

[B37] Magnetto S, Boissier S, Delmas PD, Clezardin P (1999). Additive antitumor activities of taxoids in combination with the bisphosphonate ibandronate against invasion and adhesion of human breast carcinoma cells to bone.. Int J Cancer.

[B38] Jagdev SP, Coleman RE, Shipman CM, Rostami H, Croucher PI (2001). The bisphosphonate, zoledronic acid, induces apoptosis of breast cancer cells: evidence for synergy with paclitaxel. Br J Cancer.

[B39] Stearns ME, Wang M (1996). Effects of alendronate and taxol on PC-3 ML cell bone metastases in SCID mice. Invasion Metastasis.

[B40] Hortobagyi GN, Theriault RL, Porter L, Blayney D, Lipton A, Sinoff C, Wheeler H, Simeone JF, Seaman J, Knight RD (1996). Efficacy of pamidronate in reducing skeletal complications in patients with breast cancer and lytic bone metastases. Protocol 19 Aredia Breast Cancer Study Group. N Engl J Med.

[B41] Diel IJ, Solomayer EF, Costa SD, Gollan C, Goerner R, Wallwiener D, Kaufmann M, Bastert G (1998). Reduction in new metastases in breast cancer with adjuvant clodronate treatment.. N Engl J Med.

[B42] Lipton A (2000). Bisphosphonates and breast carcinoma: present and future. Cancer.

[B43] Ellis WJ, Vessella RL, Buhler KR, Bladou F, True LD, Bigler SA, Curtis D, Lange PH (1996). Characterization of a novel androgen-sensitive, prostate-specific antigen-producing prostatic carcinoma xenograft:  LuCaP 23.. Clin Cancer Res.

[B44] Tiffany NM, Wersinger EM, Garzotto M, Beer TM (2004). Imatinib mesylate and zoledronic acid in androgen-independent prostate cancer. Urology.

[B45] Saad F (2002). Zoledronic acid significantly reduces pathologic fractures in patients with advanced-stage prostate cancer metastatic to bone. Clin Prostate Cancer.

[B46] Fizazi K, Sikes CR, Kim J, Yang J, Martinez LA, Olive MC, Logothetis CJ, Navone NM (2004). High efficacy of docetaxel with and without androgen deprivation and estramustine in preclinical models of advanced prostate cancer. Anticancer Res.

[B47] Kim SJ, Uehara H, Yazici S, He J, Langley RR, Mathew P, Fan D, Fidler IJ (2005). Modulation of bone microenvironment with zoledronate enhances the therapeutic effects of STI571 and paclitaxel against experimental bone metastasis of human prostate cancer. Cancer Res.

